# The efficacy of metal nanocomposite (Fe_3_O_4_/CuO/ZnO) to ameliorate the toxic effects of ochratoxin in broilers

**DOI:** 10.1186/s12917-022-03400-7

**Published:** 2022-08-15

**Authors:** Nagla F. Al Shap, Eman M. El. El-Sherbeny, Dalia M. A. El Masry

**Affiliations:** 1 Toxicology Unit Animal Health Research Institute, Tanta lab.Agricultural Research Center (ARC), Giza, Egypt; 2 Pharmacology Unit Animal Health Research Institute, Tanta lab. Agricultural Research Center (ARC), Giza, Egypt; 3 Nanomaterials Research and Synthesis Unit, Animal Health Research Institute, Agricultural Research Center (ARC), Giza, 264 Egypt

**Keywords:** Metal nanocomposite Fe_3_O_4_/CuO/ZnO, Chicken body weight, Total leucocytic count, Metal and ochratoxin-residue, Liver and kidney function

## Abstract

**Background:**

The study aimed to investigate the effectiveness of different doses of metal nanocomposite (MNc) (Fe_3_O_4_/CuO/ZnO) lower than its cytotoxic level in order to overcome or minimize the ochratoxin (OTA) adverse effects in broilers fed on contaminated ration.

The study conducted on 120 one-day old chicks which were divided into equal 6 groups; G1: negative control, G2: positive control (fed on OTA 17 ppb), G3& G4 (fed MNc only with low and high doses respectively). The rest two groups G5 & G6 (treatment groups) were fed on OTA, post induced ochratoxification, treated with low and high doses respectively.

**Results:**

Body weight gain and heamatocellular elements in both treated groups increased significantly than control. Serum phagocytic nitric oxide levels were increased significantly in both treated groups than control groups.

Prothrombin time (PT), Alanine aminotransferase (ALT) and gamma-glutamyltransferase (GGT) activities decreased significantly (*P* < 0.05) in both treated groups than intoxicated control group (G2) but still higher than non-intoxicated control group (G1). Total protein, albumin, globulin, calcium and phosphorus increased significantly in both treated groups than intoxicated control group. Kidney function tests showed significant improvement in both treated groups than intoxicated control group.

Antioxidant study revealed that malondialdehyde (MDA) decreased significantly in treated groups than intoxicated control group. Ochratoxin residue decreased significantly in treated groups. Metal residues in tested liver and muscle of treated groups showed no-significant difference with non-intoxicated control group (G1) at the experiment’s end.

In conclusion, feeding either low or high doses of MNc to broilers were significantly counteracting the negative impacts of OTA or its residue and increase their body weight.

## Background

Ochratoxins A, B, C are mycotoxins secondary metabolite forms [1] produced by filamentous fungi; *Aspergillus* and *Pencillium* sp. under moderate environmental conditions, where ochratoxin (OTA) is the most prevalent and relevant fungal toxin causing nephrotoxic, hepatotoxic, cytotoxic, immune suppressant besides decreased productivity in poultry [2], potent teratogenic, carcinogenic, mutagenic in human [3] and has endocrine-disrupting effects to all animal species [4, 5]. It is of great hazard that it accumulates in farm animal’s tissues, milk or poultry meat and eggs as toxic residues threaten human consumers [3, 6] resulting in nephrotoxic or carcinogenic factors [2]. Detoxification of OTA contaminated human food (poultry meat) is of global target tried by bentonite, activated charcoal [7], modified clinoptilolite based adsorbent [8], probiotics [9], or yeast and lactic acid bacteria [10]. Nowadays, through biotransformation of mycotoxins into less toxic metabolites, genetic improvement and application of nanotechnology proved tremendous potential in reducing mycotoxin production [3].

Nanobiotechnology is a novel promising innovative solution, effective, eco-friendly and low-cost strategy specially the uniqueness of metal oxide NPs (MONPs) and their ability to manufacture in a large scale due to their physicochemical properties; high surface area, very tiny size, enhanced reactivity and strong adsorbing ability. They are less toxic than salts of the same metals with a prolonged effect on biological objects [11, 12].

Regarding to biomedical and biological applications, FeONPs has efficient intracellular delivery that enhance the therapeutic agent efficacy, low toxicity, biocompatibility, general physical and chemical stability in air and their ability to be degraded or metabolized in vivo, inexpensive to produce, and environmentally safe etc. [13]. CuONPs are relatively more bioavailable, exhibited beneficial effects on immunological parameters, improved growth performance, decreased pathogenic load to subsequently enhance the health of birds [12, 14]. ZnONPs may be used at lower doses, enter the intestinal cells and grant more beneficial effects, highly bioavailable, exerting a superior efficacy and bioactivity than conventional ZnO [15].

Many authors reviewed the in vitro application of nanomaterials like silver (AgNPs), copper (CuNPs), zinc oxide (ZnONPs) in binding and removing mycotoxins or pathogens from human food [16] and animal’s feedstuffs [17] with optimal hydrodynamic diameter (1–100 nm) that affects the broad practice of their in vivo application in medicine and biology [18] besides their positive impacts on performance [19]. For ZnONPs and Fe_2_O_3_NPs only their antifungal availability is evidenced but their antimycotoxins effect was limited [20]. Both Fe_2_O_3_ and Fe_3_O_4_ NPs have antifungal activity where Fe_2_O_3_ is more than Fe_3_O_4_ [21].

The novelty of our study reputable from considering the physicochemical features of NPs and their mode of action in ochratoxicosis pathophysiology into account that assist in optimizing therapeutic benefits, improved targeting and safeguard their commercial application in the poultry industry. Hence, more in vivo studies were needed to examine therapeutic efficacy, immunogenic properties, and side effects besides the main relationship between the used doses of these NPs and corresponding occurred residues accumulation or cytotoxicity to achieve to the safest effective dose for treatment. Many studies previously pointed out the nutritional importance of each separated Fe_3_O_4_, CuO or ZnO NPs as supplementations in poultry feeding. Since, there is very limited available information on the ameliorating effects of potentiated metal oxide nanocomposite on ochratoxin in in vivo and no available literatures dealt with their synergistic action, the present study tried as the first approach to evaluate the efficacy of balanced metal nanocomposite (MNc) (Fe_3_O_4_/CuO/ZnO) at both low and high doses less its cytotoxic level to exclude the toxic effects of OTA in broilers and improve their general health status by monitoring its effect on body weight, hematological, immunological and biochemical parameters; activities of metabolic enzymes, antioxidant and lipid peroxidation in addition to determine Fe, Cu and Zn tissue residues and their effect on OTA residues in liver and muscle for consumer health.

## Material and methods

### Preparation of Fe_3_O_4_/CuO/ZnO nanocomposite

Using modified method of Velmurugan et al. [22], the magnetization of substituted ferrite NPs were prepared by coprecipitating aqueous solutions of CuCl_2_, ZnCl_2_ and FeCl_3_ (Sigma-Aldrich Co.) mixtures respectively in alkaline medium (NaOH; SigmaAldrich Co.) done in Nanomaterials Research and Synthesis Unit, AHRI, Egypt.

### Characterization of metal nanocomposite

Zetasizer Malvern Instrument (Corp, Malvern, UK) was used to measure droplet size, surface charge (zeta potential), size distribution (polydispersity indexes PDI) and electrical conductivity of the NPs. Highresolution transmission electron microscopy (HRTEM) observations were performed with a JEM 1400F HRTEM at beam energy of 300 keV. Inductively coupled plasma mass spectrometry (ICP-MS) wasused to detect the concentration of metals in nanocomposite by using the Thermo Scientific™ Qtegra™ Intelligent Scientific Xray Diffraction (XRD) according to Wright et al. [23], to use information about the position, intensity, width, and shape of diffraction peaks in a pattern from a polycrystalline sample.

### Cytotoxicity assay

Cell viability was assessed by sulforhodamine B (SRB) assay with different concentrations (0.01, 0.1, 1, 10 and 100 μg/ml) according to Allam et al. [24].

### Cell culture

Cells were maintained in DMEM media supplemented with 100 mg/ml of streptomycin, 100 units/ml of penicillin and 10% of heat-inactivated fetal bovine serum in humidified, 5% (v/v) CO_2_ atmosphere at 37 °C. Vero Green monkey cell line was obtained from Nawah Scientific Inc., (Mokatam, Cairo, Egypt).

### Ration analysis and source of ochratoxin

Aflatoxin and ochratoxin levels in the used commercial rations were analyzed by the Fluorometer (Vicam series-4) as part per billion (ppb = μg/kg) in Fungal and Mycotoxin unit, AHRI, Tanta lab., using immune affinity method of Truckess et al. [25] and Scott and Kanhere [26] respectively.

OTA free groups were fed on commercial ration free from the tested mycotoxins, while other OTA fed groups were fed on commercial ration with 2-ppb aflatoxin and 17-ppb ochratoxin (AHRI) that consider less than the permissible limit of aflatoxin and more than that of ochratoxin according to Codex [27], EC [28–30] and EFSA et al. [31].

### Broilers experimental design

A total of 120 1 day old broiler chicks were divided into 6 equal groups (20 birds/group), where the experiment was designed to allow birds free access to the prepared rations. Both groups 1&2 were control groups where, G1 was considered as control negative which did not receive any of OTA or MNc all over the experiment and G2 (OTA group) which fed (17 ppb) as control positive till the end of the experiment.

Groups 3&4 were fed only MNc from day 18 up to day 28 of age since, G3 received low dose of MNc (0.5 g/10 litter) and G4: received high dose (1 g/10 litter). Birds of the last two groups (G5 & G6; treatment groups) received OTA from the beginning of the experiment up to day 18 of age, then G5 were fed on MNc with low dose (0.5 g/10 litter) and G6 received high dose of MNc (1 g/10 litter) up to another 10 days (day 28).

All groups had the same management and vaccination with free access to ration and water. Chickens of each group were weighted at one-day-old then weekly until the end of the study up to day 35 of age.

The animal studies were approved by the Institutional Animal- Care and Use- Committee (ARC-IACUC) at Agricultural Research Center is organized and operated according to the world Organization for Animal Health (OIE) and the Eighth Edition of the Guide for the Care and Use of Laboratory Animal (2011). (ARC-AH-19/33).

## Sampling

### Blood samples

From all groups, blood samples were collected through bird wing vein puncture twice all over the experimentat the 28th and 35th day of age corresponding to post treatment-(PT) and at the end of the study respectively, as 5 birds from each experimental group. From each of them, four blood samples were collected and subjected as follow: the first one was prepared for hematological evaluation using a drop of 10% soluble EDTA for manual total leucocytic count (T.L.C) Feldman et al. [32].

Another drop which was spread and stained with Giemsa for differential leucocytic count (D.L.C) according to Campbell [33].

The second sample was collected on 1 part of trisodium citrate (3.2%) for determination of prothrombin time Quick, [34], while the third sample was collected heparinized for assaying the immunomodulatory activity (macrophage phagocytic activity expressing nitric oxide (NO)) Rajarman et al. [35] and Municio et al. [36].

The last fourth sample (the greatest one volumetrically) was collected and left to clot, then centrifuged at 3000 rpm/10 minutes where the obtained serum was prepared for biochemical analysis as for liver function evaluation; alanine aminotransferase (ALT) activity [37], gamma glutamyl transferase (GGT) which is the marker of amino acid balance [38, 39], total protein (TP) [40] and albumin [41], then, globulin level could be calculated according to Coles [42]. As well as, calcium [43] and inorganic phosphorus [44] were detected. For antioxidants assaying; malondialdehyde (MDA) [45] and reduced glutathione (GSH) [46], while for kidney function evaluation, uric acid [47] and creatinine [48] were estimated. All testes were determined using commercial kits (Spectrum and Biodiagnostic, Egypt).

### Tissue samples

Tissue specimens were collected twice all over the experiment at the 28th and 35th day of age as 5 birds from each experimental group. Post sacrificing them, liver, breast and thigh muscles samples were collected for detection of Fe, Cu and Zn residues in Animal Health Research Institute using atomic absorption spectrophotometer according to NMLK-AOAC Official Method [49].

Ochratoxin residues measured in muscle and liver at the end of the study using Symmetric technology lateral flow assay kit (ProGnosis Biotech, Larissa, Greece) and S Flow reader operated with the Lateral Logic software to quantify results in (ppb) using specific curves to calculate the results [50, 51].

### Statistical analysis

Experimental data were assessed by one-way analysis of variance (ANOVA). Duncan Multiple Range test using IBM SPSS software statistical program (windows version 20.0, USA) was used for comparison of means (*P* < 0.05). Data were expressed as mean ± SE (standard error) when (*N* = 5).

## Results

### Characterization of (Fe_3_O_4_/CuO/ZnO)-nanocomposite

**Fig. 1:**
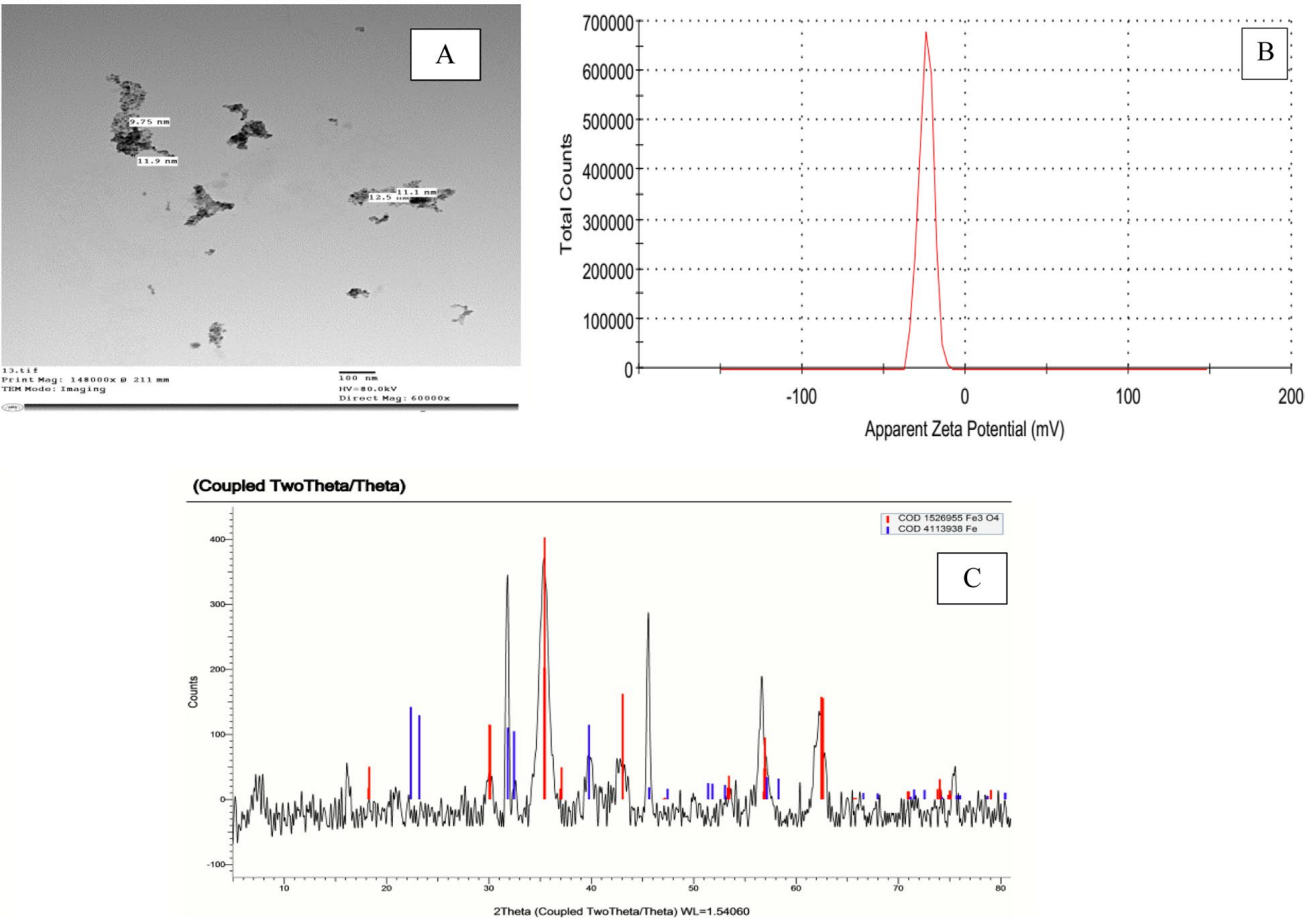
**A** TEM of (Fe_3_O_4_/CuO/ZnO)-Nanocomposite showed 10.15 ± 0.66 nm sphere shape and no aggregation. **B** Zeta potential intensity distributions for (Fe_3_O_4_/CuO/ZnO) Nanocomposite. **C** XRD pattern of (Fe_3_O_4_/CuO/ZnO) Nanocomposite

Regarding to particle size, morphology and size distribution, TEM was used for determining the size and morphology of (Fe_3_O_4_/CuO/ZnO) nanocomposite. It showed no aggregation and narrow size distribution 10.15 ± 0.66 nm with polydispersity index (PdI):0.408 ± 0.07 indicating the great homogeneity, which can be realized as shown in Fig. 1A. The zeta potential is an indicator for unstable and stable suspensions. It is generally taken by using dynamic light scattering (DLS). The zeta potential results illustrated that nanocomposite had a 23.86 ± 4.47 mV, conductivity 0.0973 ms/cm measured at 5 pH as in Fig. 1B. The concentration of (Fe_3_O_4_/CuO/ZnO)-nanocomposite in final product Fe was 47.1, Cu 6.75, and Zn 7.2 (%) measured by ICP-MS in Central laboratory at Ain Sham University, Egypt. XRD confirmed that Fe_3_O_4_ was hexagonal crystalline with 75.89% of nano elements present in the Metal composites as in Fig. 1C.

### Cytotoxicity

**Fig. 2:**
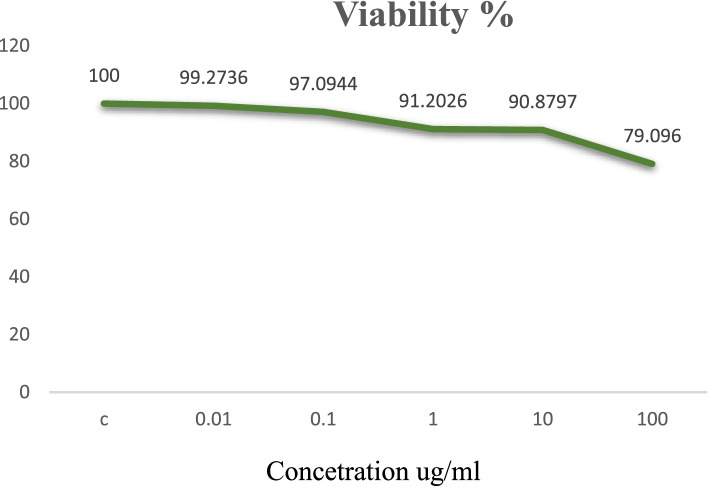
Cell viability% was assessed by SRB-assay

On the confluent surface of Vero cells, specific concentrations of (Fe_3_O_4_/CuO/ZnO)-Nanocomposite (0.01, 0.1, 1, 10 and 100-μg/ml) were inoculated. The effect on cell viability % was assessed after 3 days of inoculation by SRB-assay to be 99.2 ± 0.41, 97.09 ± 0.97, 91.2 ± 1.6, 90.87 ± 1.79 and 79.09 ± 1.58 respectively as shown in Fig. 2.

### Clinical signs and mortality

OTA fed G2, G5 and G6 showed various clinical signs and behavioral alterations as anorexia, depression, increased water intake, diarrhea and ruffled feathers where severity of signs in G2 increased gradually throughout the experimental period. No mortalities were recorded throughout the study. In treated-groups both doses relieved severity of signs in various degrees.

### Body weight

In Table 1, body weights of OTA fed G2, G5 and G6 during the first 3 weeks were significantly lower than OTA free G1, G3 and G4. No significant difference was recorded among all OTA free groups throughout the study, while a significant increase was recorded in OTA treated (G5 and G6) than G2.

**Table 1:** Effect of low and high doses of metal nanocomposite on body weight (g) (Mean ± SE) *n* = 5

Group	Initial weight	1st week	2nd week	3rd week	4th week	5th week
**G1**	45.40 ± 1.14^a^	197.20 ± 4.19^a^	494 ± 5.10^a^	961 ± 6.40^a^	1540 ± 15.49^d^	2200 ± 18.60^c^
**G2**	45.60 ± 1.03^a^	166 ± 5.10 b	400 ± 4.47^b^	872 ± 9.17^b^	1284 ± 8.12^a^	1834 ± 22.49^a^
**G3**	45.40 ± 0.81^a^	191.80 ± 5.85^a^	504 ± 11.66^a^	935 ± 6.73^a^	1576 ± 10.77^de^	2250 ± 21.46^c^
**G4**	45.20 ± 0.98^a^	199 ± 6.40^a^	501.60 ± 7.39^a^	950 ± 10.49^a^	1588 ± 10.20^e^	2244 ± 17.68^c^
**G5**	45.40 ± 1.05^a^	166.80 ± 4.44^b^	390 ± 4.47^b^	870 ± 9.49^b^	1362 ± 12.41^b^	1928 ± 13.56^b^
**G6**	45.50 ± 1.12^a^	169.80 ± 4.59^b^	382 ± 5.83^b^	860 ± 8.94^b^	1416 ± 12.27^c^	1924 ± 12.05^b^

The various letters in the same column indicate statistically significant differences when-(*P* < 0*.*05)

G1 = OTA free control, G2 = OTA fed positive control, G3 = OTA free (low dose), G4 = OTA free (high dose), G5 = OTA treated (low dose), G6 = OTA treated (high dose)

### Hematological and immunological evaluations

#### T.L.C and D.L.C

In Table 2 after treatment, T.L.C, heterophils (H) and monocytes (M) counts of OTA fed G2 showed a significant decrease when compared to OTA free G1, while OTA free (G3 and G4) showed a significantincrease in T.L.C and M count than G1. In OTA-treated-(G5 and G6) showed asignificant increase in T.L.C, H and M counts than both controls. No significant difference was recorded among all groups in lymphocyte count. At the end of the study G2 continued its decrease in T.L.C, H and M counts than G1, while treated-groups with or without OTA showed no significant difference with G1.

**Table 2:** Effect of low and high doses of metal nanocomposite on total leucocytic count (T.L.C) × 10^3^/ul and differential leucocytic count (D.L.C): monocytes (M), lymphocytes (L), heterophils (H), eosinophils (E) and basophils (B) (% of T.L.C), phagocytic activity of macrophage expressed as nitric oxide (NO) level and prothrombin time (PT) (Mean ± SE) *n* = 5

	G	T.L.C × 10^**3**^ /ul	M%	L%	H %	E%	B%	PhagocyticNO conc (μm)	PT (Second)
**After treatment**	**1**	13.87 ± 0.08^b^	4.00 ± 0.00^b^	55.60 ± 0.67^a^	35.40 ± 0.60^a^	5.00 ± 0.00^a^	0.00 ± 0.00^a^	82.80 ± 2.60^b^	11.33 ± 0.2^ab^
	**2**	12.67 ± 0.09^a^	2.00 ± 0.00^a^	54.40 ± 0.60^a^	31.00 ± 0.80^b^	9.60 ± 0.40^b^	3.00 ± 0.00^b^	65.20 ± 1.60^a^	17.33 ± 0.40^e^
	**3**	14.60 ± 0.06^c^	5.80 ± 0.20^bc^	54.60 ± 1.20^a^	35.00 ± 1.40^a^	4.00 ± 0.00^a^	0.60 ± 0.00^c^	89.20 ± 2.00^bc^	11.00 ± 0.00^a^
	**4**	15.73 ± 0.08 d	6.60 ± 0.20^c^	55.60 ± 1.20^a^	36.00 ± 0.60^a^	1.60 ± 0.00^c^	0.20 ± 0.00^a^	108.20 ± 3.80^d^	12.33 ± 0.20^b^
	**5**	15.70 ± 0.12^d^	7.00 ± 0.40^c^	54.40 ± 0.60^a^	37.60 ± 0.80^c^	1.00 ± 0.00^c^	0.00 ± 0.00^a^	98.40 ± 2.80^cd^	15.67 ± 0.20^d^
	**6**	15.97 ± 0.08^d^	7.60 ± 0.40^d^	54.40 ± 0.33^a^	38.00 ± 0.60^c^	0.00 ± 0.00^d^	0.00 ± 0.00^a^	102.20 ± 3.20^d^	14.33^c^ ± 0.40
**At the end of the experiment**	**1**	14.30 ± 0.21^c^	4.60 ± 0.00^a^	56.40 ± 0.58^a^	35.00 ± 1.20^a^	4.00 ± 0.00^a^	0.00 ± 0.00^a^	157.40 ± 3.80^d^	12.33 ± 0.40^a^
	**2**	11.07 ± 0.19^a^	2.20 ± 0.00^b^	54.60 ± 0.60^a^	32.60 ± 0.80^b^	8.60 ± 0.40^b^	2.00 ± 0.00^b^	96.40 ± 3.80^a^	19.67 ± 0.40^c^
	**3**	14.00 ± 0.15^bc^	4.60 ± 0.20^a^	56.00 ± 0.40^a^	36.40 ± 0.40^a^	2.00 ± 0.00^c^	1.00 ± 0.00^c^	123.60 ± 2.60^b^	12.00 ± 0.00^a^
	**4**	13.87 ± 0.18^bc^	4.00 ± 0.00^a^	55.60 ± 0.40^a^	36.40 ± 0.60^a^	4.00 ± 0.00^a^	0.00 ± 0.00^a^	129.80 ± 3.40^b^	11.66 ± 0.40^a^
	**5**	13.70 ± 0.12^b^	3.40 ± 0.00^b^	57.20 ± 0.60^b^	35.40 ± 0.60^a^	3.00 ± 0.00^a^	1.00 ± 0.00^c^	105.00 ± 2.80^a^	15.33 ± 0.40^b^
	**6**	14.03 ± 0.19^bc^	4.00 ± 0.00^a^	55.40 ± 0.60^a^	36.00 ± 0.60^a^	3.60 ± 0.00^a^	1.00 ± 0.00^c^	146.72 ± 1.80^c^	14.00 ± 0.40^b^

The various letters in the same column of the same period indicate statistically significant differences when (*P* < 0*.*05)

G1 = OTA free control, G2 = OTA fed positive control, G3 = OTA free (low dose), G4 = OTA free (high dose), G5 = OTA treated (low dose), G6 = OTA treated (high dose)

#### Phagocytic NO. level

After treatment, in Table 2, G2 showed a significant decrease than G1. In OTA free G4 (high dose) showed a significant increase, while G3 (low dose) showed no significant difference when compared to G1. Both OTA treated (G5 and G6) showed a significant increase than both controls. At the end, G2 continued its decrease than G1, while OTA-treated G6 (high dose) still has a significant increase than G2.

### Prothrombin time (PT)

After treatment and at the end in Table 2, no significant difference was recorded among G1, G3, and G4. G2 showed asignificant increase than G1 indicating more time needed blood to clot. OTA treated (G5 and G6) showed asignificant decrease than G2 but still higher than G1.

#### Biochemical parameters

In Table 3 results of TP, albumin, globulin and A/G ratio after treatment and at the end showed that OTA fed G2 had a significant decrease, while no significant difference was recorded among OTA free (G3, G4) and G1. OTA treated (G5 and G6) showed a significant increase than G2.

**Table 3:** Effect of low and high doses of metal nanocomposite on total protein, albumin (A), globulin (G), A/G ratio, alanine aminotransferase (ALT) and gamma glutamyl transferase (GGT) (Mean ± SE) *n* = 5

	G	Total protein (g/dl)	Albumin (g/dl)	Globulin (g/dl)	A/G ratio	ALT (u/l)	GGT (u/l)
**After treatment**	**1**	3.42 ± 0.10^c^	1.64 ± 0.05^b^	1.78 ± 0.04^c^	0.92 ± 0.03^a^	4.00 ± 0.11^a^	12.56 ± 0.44^a^
	**2**	2.43 ± 0.07^a^	1.33 ± 0.02^a^	1.1 ± 0.02^a^	1.21 ± 0.05^c^	5.83 ± 0.06^b^	14.00 ± 0.64^b^
	**3**	3.34 ± 0.11^c^	1.45 ± 0.03^ab^	1.89 ± 0.02^c^	0.77 ± 0.02^a^	5.33 ± 0.13^b^	13.60 ± 0.32^b^
	**4**	3.57 ± 0.13^c^	1.46 ± 0.04^ab^	2.11 ± 0.05^c^	0.69 ± 0.02^a^	6.53 ± 0.12^c^	14.65 ± 0.27^c^
	**5**	2.85 ± 0.09^b^	1.48 ± 0.03^ab^	1.37 ± 0.03^b^	1.08 ± 0.03^b^	6.80 ± 0.17^c^	14.53 ± 0.45^c^
	**6**	2.96 ± 0.06^b^	1.52 ± 0.05^b^	1.44 ± 0.03^b^	1.06 ± 0.03^b^	7.30 ± 0.30^d^	15.32 ± 0.68^d^
**At the end of the experiment**	**1**	3.13 ± 0.14^b^	1.70 ± 0.07^b^	1.43 ± 0.04^b^	1.19 ± 0.05^a^	4.43 ± 0.09^a^	14.33 ± 0.41^a^
	**2**	1.97 ± 0.08^a^	1.23 ± 0.04^a^	0.74 ± 0.02^a^	1.66 ± 0.05^b^	6.02 ± 0.14^c^	17.66 ± 0.57^c^
	**3**	2.98 ± 0.12^b^	1.47 ± 0.05^ab^	1.51 ± 0.06^c^	0.97 ± 0.01^a^	4.89 ± 0.07^a^	13.00 ± 0.34^a^
	**4**	3.10 ± 0.08^b^	1.56 ± 0.04^b^	1.54 ± 0.05^c^	1.01 ± 0.03^a^	5.45 ± 0.11^b^	15.32 ± 0.46^b^
	**5**	2.83 ± 0.07^b^	1.45 ± 0.06^ab^	1.38 ± 0.05^b^	1.05 ± 0.02^a^	5.62 ± 0.11^b^	15.00 ± 0.52^b^
	**6**	2.98 ± 0.07^b^	1.61 ± 0.07^b^	1.37 ± 0.04^b^	1.18 ± 0.04^a^	5.87 ± 0.10^c^	17.78 ± 0.63^c^

The various letters in the same column of the same period indicate statistically significant differences when (*P* < 0*.*05)

G1 = OTA free control, G2 = OTA fed positive control, G3 = OTA free (low dose), G4 = OTA free (high dose), G5 = OTA treated (low dose), G6 = OTA treated (high dose)

Regarding to activities of ALT and GGT after treatment and at the end, G2 and all treated groups showed significant increase than G1. Meanwhile, low doses caused less increase than high doses.

In Table 4 results of Ca and Ph levels after treatment and at the end, showed that OTA-fed-G2 decreased significantly than G1 while no significant difference was recorded among OTA free (G3, G4) and G1. OTA-treated-(G5 and G6) showed a significant increase than G2.

**Table 4:** Effect of low and high doses of metal nanocomposite on cacium (Ca), phosphorus (Ph), malondialdehyde (MDA), reduced glutathione (GSH), uric acid and creatinine (Mean ± SE) *n* = 5

	G	Ca (mg/dl)	Ph (mg/dl)	MDA (nmol/ml)	GSH (mg/dl)	Uric acid (mg/dl)	Creatinine (mg/dl)
**After treatment**	**1**	7.98 ± 0.14^b^	7.56 ± 0.15^b^	3.40 ± 0.09^a^	0.28 ± 0.01^b^	6.85 ± 0.19^b^	0.32 ± 0.01^a^
	**2**	6.02 ± 0.12^a^	6.02 ± 0.11^a^	8.95 ± 0.27^c^	0.16 ± 0.00^a^	7.83 ± 0.23^c^	0.56 ± 0.02^c^
	**3**	7.87 ± 0.10 b	7.34 ± 0.21^b^	4.77 ± 0.12^a^	0.35 ± 0.00^d^	6.02 ± 0.17^a^	0.28 ± 0.00^ab^
	**4**	8.11 ± 0.14^b^	7.65 ± 0.18^b^	6.81 ± 0.25^b^	0.36 ± 0.01^d^	6.51 ± 0.10^a^	0.36 ± 0.01^a^
	**5**	6.65 ± 0.09^a^	6.87 ± 0.12^b^	7.24 ± 0.24^b^	0.32 ± 0.00^c^	7.29 ± 0.12^b^	0.45 ± 0.01^ab^
	**6**	7.43 ± 0.13^b^	7.37 ± 0.10^b^	6.59 ± 0.19^b^	0.36 ± 0.01^d^	7.64 ± 0.26^b^	0.50 ± 0.02^a^
**At the end of the experiment**	**1**	7.27 ± 0.31^b^	8.18 ± 0.30^bc^	4.20 ± 0.17^a^	0.31 ± 0.01^b^	7.00 ± 0.25^b^	0.37 ± 0.01^ab^
	**2**	5.47 ± 0.12^a^	6.16 ± 0.15^a^	9.08 ± 0.31^c^	0.19 ± 0.00^a^	9.33 ± 0.31^c^	0.62 ± 0.02^c^
	**3**	7.30 ± 0.10^b^	7.87 ± 0.19^b^	4.14 ± 0.13^a^	0.30 ± 0.01^b^	4.40 ± 0.12^a^	0.33 ± 0.01^ab^
	**4**	7.43 ± 0.27^b^	8.38 ± 0.26^bc^	5.11 ± 0.21^ab^	0.35 ± 0.01^c^	5.63 ± 0.18^a^	0.43 ± 0.00^a^
	**5**	6.50 ± 0.11^b^	7.90 ± 0.22^b^	6.54 ± 0.26^b^	0.29 ± 0.01^b^	6.17 ± 0.24^b^	0.33 ± 0.01^ab^
	**6**	7.03 ± 0.24^b^	8.37 ± 0.28^bc^	5.53 ± 0.15^b^	0.35 ± 0.01^c^	6.73 ± 0.21^b^	0.53 ± 0.00^a^

The various letters in the same column of the same period indicate statistically significant differences when (*P* < 0*.*05)

G1 = OTA free control, G2 = OTA fed positive control, G3 = OTA free (low dose), G4 = OTA free (high dose), G5 = OTA treated (low dose), G6 = OTA treated (high dose)

MDA levels after treatment and at the end, revealed that no significant difference between G1 and G3. OTA treated (G6 followed by G5) showed a significant decrease than G2 while G2 showed a significant increase than all groups throughout the study. Regarding to GSH after treatment and at the end, OTA free (G3 and G4) showed a significant increase than G1 with no significant difference between each other. OTA treated (G6 followed by G5) showed a significant increase than G2 while G2 showed a significant decrease than all groups throughout the study.

Uric acid and creatinine levels after treatment and at the end revealed that G2 showed a significant increase while there was a significant decrease in both OTA free (G3 and G4) than G1 in uric acid and no significant difference among them in creatinine. OTA treated (G5 and G6) showed a significant decrease than G2 and no significant difference when compared with G1. Meanwhile, low doses caused more decrease than high doses.

#### Residues of metal nanocomposite in liver and muscles

In Table 5 results of Fe residues in liver showed a significant decrease in OTA fed G2 than OTA free G1. After treatment, only high doses (G4 and G6) were significantly more than both controls, while at the end no significant difference was recorded among them and G1. Fe residues in muscle showed a significant increase in G2 than G1. Throughout the experiment high doses (G4 and G6) were significantly lower than G1.

**Table 5:** Effect of low and high doses of metal nanocomposite on iron (Fe), copper (Cu) and zinc (Zn) residues in liver and muscle (Mean ± SE) *n* = 5

	G	Liver			Muscle		
		Fe (μg/ml)	Cu (μg/ml)	Zn (μg/ml)	Fe (μg/ml)	Cu (μg/ml)	Zn (μg/ml)
**After treatment**	**1**	22.96 ± 0.35^c^	1.50 ± 0.01^a^	8.07 ± 0.10^a^	11.92 ± 0.24^d^	0.73 ± 0.01^a^	8.54 ± 0.11^c^
	**2**	16.86 ± 0.33^a^	5.11 ± 0.08^d^	12.40 ± 0.18^c^	14.46 ± 0.20^e^	0.54 ± 0.01^a^	6.98 ± 0.08^b^
	**3**	20.75 ± 0.28^b^	2.29 ± 0.03^b^	9.96 ± 0.17^b^	5.62 ± 0.07^b^	0.60 ± 0.01^a^	6.09 ± 0.28^a^
	**4**	42.17 ± 0.54^e^	3.13 ± 0.04^c^	14.63 ± 0.21^d^	8.35 ± 0.11^c^	0.97 ± 0.01^b^	7.14 ± 0.08^b^
	**5**	15.91 ± 0.25^a^	2.19 ± 0.02^b^	8.64 ± 0.25^a^	4.33 ± 0.07^a^	0.68 ± 0.02^a^	5.70 ± 0.16^a^
	**6**	33.24 ± 0.43^d^	2.26 ± 0.04^b^	9.78 ± 0.23^b^	8.44 ± 0.08^c^	1.26 ± 0.14^c^	6.09 ± 0.13^a^
**At the end of experiment**	**1**	22.03 ± 0.17^de^	1.50 ± 0.02^c^	7.84 ± 0.10^c^	11.08 ± 0.15^e^	0.61 ± 0.01^d^	7.80 ± 0.08^e^
	**2**	16.76 ± 0.14^b^	4.24 ± 0.15^d^	11.39 ± 0.18^d^	13.52 ± 0.25^f^	0.43 ± 0.01^c^	5.85 ± 0.10^c^
	**3**	13.74 ± 0.30^a^	0.74 ± 0.01^b^	5.82 ± 0.04^b^	3.54 ± 0.08^a^	0.12 ± 0.01^a^	3.72 ± 0.01^a^
	**4**	21.48 ± 0.49^d^	1.50 ± 0.01^c^	14.41 ± 0.24^e^	8.46 ± 0.13^d^	0.15 ± 0.00^b^	7.41 ± 0.12^d^
	**5**	20.19 ± 0.33^c^	0.09 ± 0.00^a^	3.34 ± 0.14^a^	6.44 ± 0.15^b^	0.11 ± 0.00^a^	4.11 ± 0.08^b^
	**6**	22.84 ± 0.46^e^	0.85 ± 0.01^b^	8.20 ± 0.12^c^	7.40 ± 0.18^c^	0.17 ± 0.00^b^	4.32 ± 0.04^b^

The various letters in the same column of the same period indicate statistically significant differences when (*P* < 0*.*05)

G1 = OTA free control, G2 = OTA fed positive control, G3 = OTA free (low dose), G4 = OTA free (high dose), G5 = OTA treated (low dose), G6 = OTA treated (high dose)

Cu residues in liver showed a significant increase in G2 than G1. After treatment, all treated groups in both OTA free and OTA fed were significantly more than G1, while at the end no significant difference was recorded among high doses (G4, G6) and G1. Cu residues in muscle showed a significant decrease in G2 than G1. After treatment, only high doses (G4 and G6) were significantly more than G1, while at the end they revealed a significant decrease than G1.

Zn residues in liver showed a significant increase in G2 than G1. After treatment, all treated groups in both OTA fed and OTA free were significantly more than G1. At the end, no significant difference was recorded among G6 (high doses) and G1 while G4 (high doses) still significantly more than G1. Zn residues in muscle showed a significant decrease in all groups than G1 throughout the experiment. All metal residues were decreased at the end of the study when compared with post treatment results.

#### Residues of OTA in liver and muscles

As in Table 6 OTA residue in both liver and muscle, samples of G2 revealed a significant increase than all treated groups, while both doses of treatment showed no significant difference than G1.

**Table 6:** The residues of ochratoxine in liver and muscle at the end of the experiment (Mean ± SE) *n* = 5

Group	Liver	Muscle
**G1:** OTA free control	1.50 ± 0.05^a^	1.51 ± 0.03^a^
**G2:** OTA fed control	3.00 ± 0.12^b^	2.76 ± 0.09^b^
**G5:** OTA treated (low dose)	1.51 ± 0.03^a^	1.53 ± 0.04^a^
**G6**: OTA treated (high dose)	1.50 ± 0.06^a^	1.50 ± 0.03^a^

## Discussion

OTA detoxication is of global concern through anti-OTA dietary supplements Li et al. [52]. Nanotechnology is now widely used throughout the pharmaceutical industry show tremendous potential in reducing mycotoxin production [3]. The biological response to MONPs in vivo systems occurs differently depending on their size, shape, purity, stability and surface properties so it is necessary to characterize their morphology. After entering into the body, NPs interact with biofluids and cell biomolecules that facilitate the physical transfer of the particles into the inner cellular structures [53]. They proved to be a promising tool as anti-OTA agents [20, 54, 55]. The present study aimed to study the potency of MNc amelioration of OTA toxification or its residues in broilers and its edible meat.

No relevant data was found on the association between nanomaterial affectivity, toxicity, and mycotoxin dose so, for evaluation the efficiency of the used treatment, the relation between the effect of antimycotoxin NPs and the toxic effect of mycotoxins have to be carefully considered. Consequently, our study try to fill this gap included the relationship between the threat of natural concentration of OTA found through feeding commercial feeds and the effectiveness of low and high doses of metal nanocomposite-(Fe_3_O_4_/CuO/ZnO) below its cytotoxic level to control injurious effects of OTA, monitor their effect on body weight, hematological, immunological and biochemical constituents.

Through the present study, as shown in Fig. 1A & B, Fe_3_O_4_/CuO/ZnOnanocomposite characterization have distinctive and unique characters as nano-crystalline. Shinde et al. [56] found that the X-ray diffraction pattern of cobalt ferrite sample shows the reflections (220), (311), (400), (422) and (511) belonging to cubic spinel structure and SEM image is 62 nm of the average grain size. Also, Pu et al. [57] showed that Co–Cu–Zn doped Fe_3_O_4_NP shad mean size of 23 nm size with spherical-like crystals with shape. According to ZnO/CuO (5%) and ZnO/CuO (10%) nanocomposites low weight percentages, Kuznetsov et al. [58] found that the reflection peaks of CuO with low intensity and the peaks of hexagonal crystalline phase of ZnO, were observed. Other research study showed that Zn Cu chitosan nanocomposites had 16.6–24.3 nm [59].

In the present study, OTA fed birds (G2, G5 & G6) showed various clinical signs and behavioral alterations; anorexia, depression, increased water intake, diarrhea and ruffled feathers where severity of signs in G2 increased gradually throughout the experimental period. Similar signs [2, 4] were reported. In treated groups both doses relieved severity of signs in various degrees which may attribute to the improvement of general health status of birds. As well, no mortalities were recorded throughout the study due to the chronic ochratoxicosis that is so common in poultry that fed on low to moderate quantity of toxins stored rations prepared from contaminated grains [60] where dietary dose level, duration and toxin type affect mortality rate.

It is clear from Table 1, birds body weight of OTA fed (control positive) G2 was lowered significantly (*P* < 0.05) than non-intoxicated control G1 that owing to reduced feed consumption and poor feed conversion efficiency [61], reduced the intestinal nutrition absorption, suppressed the expression of proteins responsible for intestinal integrity, altered intestinal permeability and gut microbiota [6]. Moreover, OTA decreases the villi height that increases intestinal epithelial cells apoptosis [6, 62, 63], interfering absorption and assimilation. OTA also affects the intestinal inflammation pathway by decreasing the expression of some inflammation-related cytokines [64], besides the alteration of immune system that renders the gut vulnerability to infection. Body weight of both treated groups increased significantly (*P* < 0.05) than that of induced intoxificated group (G2) since MNc (Fe_3_O_4_ / CuO / ZnO) either in low (G5) or in high (G6) doses ameliorated toxic effects of OTA. As well, it may be attributed to their positive effect on the activity of the intestinal microbiome ecosystem [65, 66], that play an important role in body physiological processes, metabolism, immune system by inducing cytokines [67], enteric homeostasis, synthesis of essential substances as vitamins [68], regulating the composition of endogenous losses of mineral substances and increasing the productivity of poultry [69]. It was established that body weight significantly increased on feeding Fe_3_O_4_NPs via improved the final weight and increased bioavailability of trace minerals which enhanced health [70–72], CuNP [14, 73] via reduced the pathogenic load that subsequently enhanced the birds’ health, improved the feed intake and nutrient digestibility (particularly protein digestion), due to easier uptake of NPs through the villus epithelium passing GIT barrier that allows them to enter directly the bloodstream and metabolized in the liver and spleen, besides significantly improved aminopeptidases activity that is vital for protein digestion in muscles [74], while in ZnONP via enhanced IL-1β, IL-10 and TNF-α, in ileal mucosa, and increased villous height, width, crypt depth, and surface area [75], SNP [19], Cu and Zn NP which resulted in a study [39] in a positive productive effect and muscle tissue by increased bioavailability of the assessed microelements than inorganic form of mineral supplements.

The studied haematological and immunological parameters revealed that birds had OTA feeding only G2 showed significant reduction than G1 in T.L.C, heterophils and monocytes percentages. Birds fed rations provided with MNc (groups G3, G4, G5 and G5) either fed OTA or not showed a significant increase in T.L.C, H and M counts, while no significant difference was recorded in lymphocytes count among all groups (Table 2). Where leukocytes responsible for providing body protection, T.L.C and D.L.C describe the health status, immune system and evaluate the effect of drugs. Monocytes and granulocytes are the first line of defense because they migrate to the site of inflammation and responsible for pathogens’ phagocytosis [76]. As well, heterophils produced bioactive molecules for pathogen recognition and destruction, cellular communication and activation; initiated an adaptive immune response and later, resoluted inflammatory responses, tissue repair and phagocytosis [77]. The immunosuppressive activity of OTA is accompanied by depression of antibody responses and immune cells expressed as a significant leukocytopenia, lymphocytopenia and monocytopenia besides the modulation of cytokines production [53], but with the ameliorative action of MNc at optimum levels of produce better results on both T.L.C and D.L.C indicating better functions of non-specific immune responses, leucocytosis with heterophilia [77–81] even in short term duration concluding that essential MONPs with their bioavailability resulted in less pronounced toxic effect.

NO in the current study which is expressed as a result of macrophage activity revealed a significant decrease in G2 (induced OTA only) than G1 (OTA free), while all birds received MNc (G3, G4, G5 and G6) showed significant increase than other both control groups as shown in Table 2. Phagocytic activity (PA) of blood macrophages enhanced immune response and disease resistance effect. These promising effects of MNc either low or high not only ameliorated the toxic effect of OTA on macrophage activity, but also modulated microphage activity even those previously intoxicated groups (G5 & G6). Increasing the expressed NO post MNc therapy is attributed to the redox-active MONPs which tend to modulate the innate and adaptive immunity and this ability which is widely documented [39, 82–84] noticed in blood cellular elements, immune organs, phagocytosis [53], as well in liver post Fe_3_O_4_NPs use [70] or CuNP as an immune system modulator [14]. Hence, reactive oxygen species (ROS) can function as a second messenger and modulator to immunity. In contrast, [85] reported that CuNP did not has the ability to stimulate the immune system, which may be due to the failure of antigen-presenting cells to recognize it, since its uptake is important in directs the immune responses. So, using more than one metal as in our study may be one of merits of using the nanocomposite (Fe_3_O_4_/CuO/ZnO) together.

PT in the present study increased significantly by the action of OTA in birds of group G2 as shown in Table 2, the results which explained the presence of hemorrhagic patches on thigh and breast muscles of broilers suffering OTA toxication. PT decreased significantly in birds of (G5 and G6) than those of G2 but still higher than those of G1. Hemostasis in chickens has received little attention although it is of importance to the poultry industry due to the basic mechanisms relate to condemnations from bruising. OTA in feedstuffs can produce serious coagulopathies in chickens as significant increases in re-calcification and PT [61] while the time required forming a fibrin clot at the higher toxin level was practically double that of the control. NPs may induce pulmonary and systemic inflammation, platelet activation, and vasomotor dysfunction [86] so in vivo studies has a critical importance. Pertaining to the effect of OTA on increasing time of blood clotting, the platelet activation of MONPs considers a good benefit.

In the present study serum TP, albumin, globulin, Ca and Ph levels revealed that no significant differences in birds fed on MNc only (G3 & G4) and those of control group (G1), while significant decrease was recorded in G2 as shown in Tables 3 and 4. OTA may integrate; inhibition of protein synthesis, disruption of cell signaling and division, impaired mitochondrial oxidation reactions and altered Ca homeostasis through suppressing the phosphoenolpyruvate carboxylase in the proximal renal tubules that alters the structural and functional renal ability to metabolize Ca [87] CuNP [73] enhances protein synthesis through utilized amino acids, improves aminopeptidases activity that is vital for protein digestion in muscles [74] and (Cu and Zn) NPs [39] have tendency to increase the content of serum protein and the activity of aminotransferases without any changes in liver microstructure. Meanwhile, low doses caused less increase in liver enzyme activities than high doses. TP and ALP was higher on feeding either Fe_3_O_4_-CysNPs ([71] or high doses Fe_2_O_3_NPs (50 mg/kg) [80] while no significant alterations in antioxidant, metabolic enzymes, and lipid peroxidation-(LP) with 10 and 20 mg/kg as safe dietary levels indicating to dose effect. The studied liver enzymes, blood samples of all birds fed on OTA, in the present study, revealed significant increase in ALT and GGT values (Table 3). Since, ALT and GGT values consider two of proper hepatic function evaluation tests hence, increased of their activities is indicative to hepatocellular injury [5]. Moreover, Balakrishna and Prabhune [88] mentioned that GGTs are highly conserved enzymes play an important role in the homeostasis of glutathione (a major cellular antioxidant) due to some of the common physiological γ-glutamyl substrates are glutathione. Furthermore, [39] consider GGT the marker of amino acid balance in the organism and assumed that the increase in ALT activity indicates changes in the metabolic flows in the treated groups, mostly for the nanoscale form. Therefore, enzymes’ activity depends on the form of mineral supplement entering the organism as regard to the effect of CuNPs. Because of OTA metabolism mainly in liver excreted through kidneys its intoxication resulted in hepatocellular injury [5] and nephrotoxic [89] even exposure to low concentration of OTA leading to morphological and functional alterations in renal and hepatic tissues thus, OTA reduced TP, albumin, globulin, Ca, Ph salts, super oxide dismutase (SOD) and glutamic peroxidase (GPX) activities and increased ALT, AST, GGT, ALP, MDA, creatinine and uric acid [89, 90]. Not only, liver and kidneys might be affected but also, gastrointestinal tract, lymphoid organs, skeletal system and hemopoietic tissues [61]. Revealed in significant increases in activities of AST, ALT, SOD, catalase and lipid peroxidation (LP) in liver and muscles by OTA, feeding on a ration provided with MNc significantly decrease in ALT, AST, and uric acid levels in the blood [73] and increase levels of serum Ca, Ph, Fe and bone resistance against fracture [91, 92]. MNc increase ALT, AST and GGT levels [93, 94] as well as creatinine [95] or decrease it [94]. Decrease of urea in plasma indicated that CuNP enhanced protein synthesis through utilized amino acids more efficiently for growth besides volume ratio of the femoral bones of chicken [73]. As well, CuNP increase TP levels and enhanced proliferating cell nuclear antigen (PCNA)-positive cells in the chicken long bones, which improved the physical characteristics of the bone [96].

As shown in the Table 4, no significant difference between G3 (MNc only fed) and G1 in MDA, where G2 (OTA only fed) showed a significant increase in MDA and decrease in GSH than all groups throughout the study. All treated groups decreased MDA significantly while increased GSH than both controls. MDA which is a stable metabolite of the free radical mediated lipid peroxidation (LP) cascade [97]. It can form complexes with other biological components such as protein, lipids, and nucleic acids while GSH is used by glutathione peroxidase in removing H_2_O_2_ generated during lipid oxidation. OTA inducted its cytotoxicity or cell injury by generation of (ROS) [52], increased their oxidative stress (OS) and lipid peroxidation (LP) that lead to the induction of numerous of cellular processes; inflammation, genetic damage, apoptosis activation particularly in renal toxicity, arresting of growth as well development of degenerative diseases including immune system suppression [98]. Negative biochemical impacts of mycotoxins affected by animal species, age, production stage, and toxin co-contamination, type, time and dose of exposure. So increasing the function of antioxidant enzymes may protect against their toxic effects [5]. The antioxidant enzymes are responsible for scavenging superoxide radicals and involved in protective mechanisms within tissue injury following oxidative process and phagocytosis. So, antioxidants aid in the overall detoxification process in the liver and in cells and thus, may aid in alleviation of mycotoxicosis. MNc acts as an effective antidote, nutritional supplements that suppress OTA toxic impacts, decrease tissue damage caused by OS, and set aside the body to sustain a viable immune system. Nevertheless, NPs absorption and metabolism depend on its dosage physicochemical properties, such as size, shape, surface chemistry and charge, length, method of administration, and dose [99]. Consequently, the benefits or drawbacks of NPS on redox reactions and immune defense depend mainly on select its effective safe dose. Similar unique antioxidant properties without any oxidative damage were recorded for Fe_3_O_4_NPs [100] CuONPs [16, 101–103] ZnONPs [39, 104, 105].

As well in (Table 4) both OTA free (G3 and G4) and OTA treated (G5 and G6) revealed a significant decrease in uric acid and creatinine levels than both control groups, while OTA fed (G2) showed a significant increase than G1. Meanwhile, low doses caused more decrease than high doses. Increased serum creatinine and uric acid concentrations indicated impaired renal function and thereby decreased glomerular filtration rate due to creatinine is freely filtered in kidney glomeruli and normally has no tubular resorption [106]. OTA is a nephrotoxin [61, 89]. For Cu, decreasing of blood urea, [107] indicated its potential association with higher protein metabolism and higher use of amino acids for protein formation. Similar findings for CuNPs [108], ZnONPs [93, 94, 109].

It is clear from Table 5 that Fe, Cu and Zn residues in liver were more than muscular specimens in all tested groups, but decreased at the end of the experiment with no significant difference than G1. It is documented [110, 111] that the elevated NPs accumulate, especially Fe, when it was singly dosed due to its high surface adsorption tendency depending on its shape, size, aggregation, and magnetic properties as important objects influencing the pharmacokinetic and biodistribution in in vivo applications determining its fate in addition to, the surface charge that plays an important role in the physical stability and influences its interaction with the biological system and their safety, while this surface adsorption mechanism may alter when Fe_3_O_4_NP combined with other NPs. So, our metal-nanocomposite has the ability to overcome the residue obstacle in both of its low or high doses refer to its balanced formation which improves its physical properties. Moreover, NPs can be rapidly cleared through the kidneys when the particle size is small [112] and this assessed in our nanocomposite characterization. The elevated Fe in studied hepatic tissues than those in muscular tissues was recorded [111], for using CuONP [102, 113].or ZnONP [109, 114–116] may be due to the high production rate of metallothionein [117] since the liver act metal detoxification through metal sulfur-protein formation [118] and storing the excess Fe form of heme-protein and ferritin for various metabolic activities [119]. The less concentration of MONPs in the muscle may be due to the low level of metal-binding protein as MT in the muscle and owing to the large mass with low metabolic activity of muscular tissues [120]. Fe_3_O_4_NP with a long blood retention time, biodegradability and low toxicity have emerged as one of the primary nanomaterials for in vivo applications. Its biodegradation metabolites related to the immune system response post its administration [72]. So, MNc in the current investigation three different metals (Fe, Cu & Zn) had the ability to overcome the residue obstacle in both of its low or high doses due to its nano-size improves its absorption and distribution in tissues.

The most positive concluded result that OTA residues either in hepatic or muscular tissue samples showed significant decrease in both treated MNc groups either of low (G5) or high (G6) dose than G2 (Table 6) and this evidenced the positive effect of MNc (Fe_3_O_4_/CuO/ZnO) in counteracts OTA residue in edible tissues as mycotoxin binders besides or overwhelming its toxic effects indicated tissue safeness to the consumer. OTA accumulates in poultry meat because they are largely lipophilic [121] as residue threaten humans consuming such meat due to its long half life, high chemical and thermal stability [6].and its presence in significant quantities poses health risks in affected humans varying from allergic reactions up to death [3, 31]. Further studies are required for investigating the synergistic effects of combined antioxidant metal-NPs and other commercial antimycotoxins to obtain dual synergistic actions, decrease chemicals amount used in feed manufacture and study in vivo availability. Up to date, [122] metal-NPs have dual synergistic effects of antioxidant, antifungal and growth promoters which will be significantly reflected in improving animal health. Even so, the possible adverse and toxicological impacts of NPs are still a matter of investigation.

## Conclusion

Consequently, despite the nanobiotechnological application in mycotoxicology is still in the early stages, it is a novel promising tool. The exclusive size-dependent properties of the NPs make these materials indispensable and superior in their activities. The present study concluded that using MNc (Fe_3_O_4_/CuO/ZnO) in both low and high in subtoxic doses were significantly counteract OTA residues and its negative impacts specially; on body weight, non-specific immune responses and overwhelming oxidative stress meanwhile, low doses caused more decrease in liver enzyme activities and improved kidney functions. New insights into advanced investigations are required on the possible mechanisms that MONPs affect OTA removal or if it act as binders in vitro or in vivo, besides their elimination efficacy against other mycotoxins.

## Data Availability

The corresponding author will provide the datasets used in this study on reasonable request.
